# An analysis of the current status of geriatric oral disease treatment—a dental institutions-based perspective

**DOI:** 10.3389/fpubh.2024.1503938

**Published:** 2025-01-14

**Authors:** Caili Li, Dongmei Huang, Qini Pan, Pinyue Tao, Xiao Pan, Yanfei Pan, Ruofei He, Tongting Wang, Huiqiao Huang

**Affiliations:** ^1^General Surgery, The Second Affiliated Hospital of Guangxi Medical University, Nanning, China; ^2^Rehabilitation Department, The Second Affiliated Hospital of Guangxi Medical University, Nanning, China; ^3^Nursing Department, The Second Affiliated Hospital of Guangxi Medical University, Nanning, China; ^4^Anesthesiology Department, The Second Affiliated Hospital of Guangxi Medical University, Nanning, China; ^5^Ear, Nose, Throat, Head and Neck Surgery, The Second Affiliated Hospital of Guangxi Medical University, Nanning, China; ^6^Orthopedic Trauma Surgery, The Second Affiliated Hospital of Guangxi Medical University, Nanning, China; ^7^Party Committee Office, The Second Affiliated Hospital of Guangxi Medical University, Nanning, China

**Keywords:** oral medical and health institutions, older adults, treatment of oral diseases, continuing medical education, current status

## Abstract

**Background:**

This study aimed to investigate and analyze the current status of oral disease treatment among the older adult in Guangxi Zhuang Autonomous Region, while also assessing the continuing medical education (CME) needs of dental institution personnel regarding oral diseases in this population.

**Methods:**

Convenience sampling was used to investigate the oral disease treatment among older adults and to assess CME needs of dental institution personnel regarding oral diseases in this population across various oral medical and health institutions in Guangxi.

**Results:**

A total of 754 valid questionnaires were collected, of which 70.3% were from non-public oral health institutions. Out-of-pocket costs for older adults were as high as 91.3%. The per capita cost of older adult patients was beyond 500 yuan in 51.6% of the oral health institutions. In terms of CME training, 32.8% of dental institution medical personnel have participated in CME courses specifically on oral diseases in the older adult. Meanwhile, 69.9% of institutions have expressed a need for CME training on oral diseases in the older adult.

**Conclusion:**

Non-public oral health institutions account for a significant proportion, and the older adult primarily pay out-of-pocket for oral disease treatment in these facilities. The high cost of treatment may pose a significant barrier to the older adult seeking oral healthcare. Increasing CME programs targeted at geriatric oral diseases can help enhance the treatment capabilities of dental healthcare workers and improve oral health outcomes for the older adult population.

## 1 Introduction

Oral disease refers to a series of chronic clinical diseases that affect teeth and the mouth, including dental caries, periodontal disease, tooth loss, and oral cancers (1). According to the World Health Organization (WHO), approximately 3.5 billion people worldwide have oral diseases, accounting for nearly half (45%) of the human population (2). Thus, making them one of the most prevalent diseases affecting human health, and a pressing public health challenge. Socioeconomic status and the accessibility and coverage of oral healthcare make poor, vulnerable, and marginalized populations the most susceptible to oral diseases (2).

As one of the vulnerable groups, this population seems to have a higher overall prevalence of oral diseases worldwide. Among older population aged 65 years and older, the average prevalence of total periodontitis was found to be 66.2% in the United States (3) and 85.7% in Turkey (4); the caries rate in Uruguay was 99.6% (5); and the prevalence of tooth loss and periodontal pockets in India have been reported to be 91.5 and 81.5%, respectively (6). According to the fourth epidemiological survey of oral health in China, periodontal health rate was 9.3%, caries rate of permanent teeth was 98%, detection rate of gingival bleeding was 82.6%, and detection rate of calculus was 90.3% among the older adults aged 65–74 years (7). A Survey report of Guangxi residents’ oral health and medical service ability found that oral problems affect the quality of life of 67.3 percent of older adults in Guangxi (8), which indicates that the oral health status of older adults is not optimistic.

Therefore, developing strategies to enhance the oral health of the older adult population is a topic warranting investigation. Previous studies have demonstrated a significant correlation between the income of older adult individuals and their utilization of oral healthcare services, with lower-income older adult being less likely to seek dental care (9, 10), highlighting economic factors as a major barrier for this group in accessing such services. Additionally, different healthcare systems have varying impacts on the access of the older adult to oral healthcare services. The National Health Service (NHS) in the UK, for example, provides free dental treatment for people aged 65 and older (11). Japan’s public health insurance covers a wide range of dental treatments, leaving only 10.8% of the population who do not use medical insurance when attending dental clinics (12). The self-funded portion accounts for approximately 10–30% of the total cost of oral diagnosis and treatment (13). Japan is therefore one of the countries with the lowest rate of dental out-of-pocket payments and the highest use of dental care, making it easier for the older adults to access oral health services.

The types of oral diseases in the older adults are complex, with atypical symptoms and signs, coexistence of multiple diseases, and other characteristics (14), which require stomatologists to make a more comprehensive assessment of the general condition of older adults and adopt safer treatment methods. However, most oral medical institutions do not have the characteristics of treatment methods for older adult patients (15). Therefore, assessing the CME needs of dentists regarding the treatment of oral diseases in the older adult is also crucial for enhancing oral healthcare services for this population.

Existing research predominantly adopts the perspective of the older adult to understand their oral health status. Furthermore, few studies have explored the CME needs of medical institution personnel regarding the treatment of oral diseases in the older adult population. Therefore, this study, grounded in the perspective of dental medical institutions, analyzes the main reasons why the older adult seek oral healthcare, their payment methods, and the economic burden they face. Additionally, it investigates the CME needs of institution personnel regarding the treatment of oral diseases in the older adult. Consequently, the study aims to explore strategies for enhancing oral healthcare services for the older adult in Guangxi Zhuang Autonomous Region by investigating and analyzing the current status of oral disease treatment among this population, while also assessing the CME needs of dental institution personnel, ultimately serving as a reference for policymakers.

## 2 Methods

### 2.1 Study settings

Guangxi Zhuang Autonomous Region at all levels and various types of oral health institutions, including dental specialty hospital, oral outpatient department, dental clinic, Department of Stomatology of General Hospital, Community Health Service Center, and Township Health Center Stomatology Department.

### 2.2 Survey tool

Based on the questionnaire regarding oral healthcare institutions from the Fourth National Oral Health Epidemiological survey report on Oral Health in 2015 (7), the questionnaire for this survey was initially designed. The research team contacted Professor Yu, of the stomatology department in a third-class A hospital, obtained his consent, and set an appointment for the interview and discussion. After two rounds of discussion, modification, and improvement with Professor Yu and his team, our team put together a questionnaire focusing on the status of oral health services for the older adults in Guangxi oral health institutions. The questionnaire included questions on (1) the nature and type of dental institutions, (2) the proportion of older adult patients treated for oral disease at dental institutions, (3) the reasons for oral disease treatment in the older adult, (4) the preferred payment method for treatment among older adult patients, (5) the expenditure for oral disease treatment among older adult patients, (6) Do medical institutions offer a specialized geriatric oral healthcare department, (7) Whether the institution’s medical staff engaged in CME regarding oral health for older adults during the previous year, (8) Does the institution have CME needs related to oral health of older adults, the term “older adult” refers to individuals aged 60 and above. To determine the internal consistency of the questionnaire, we calculated Cronbach’s *α* as 0.9, indicating a good reliability for the questionnaire.

### 2.3 Investigation method

With the assistance of the Guangxi Zhuang Autonomous Region Health Commission, the questionnaire was sent to oral health institutions of all levels and types. The questionnaire was completed by heads of the stomatology department or the oral institution and sent to the designated email address. A total of 785 questionnaires were collected, 754 of which were validated, with an effective response rate of 96.1%.

### 2.4 Quality assessment

After the questionnaire was completed, the researchers screened it. (1) Two-dimensional code questionnaire: The questionnaire with the same organization name or IP address source, retained the last answer result, if there were obvious logical errors, the questionnaire was deemed invalid. (2) Email questionnaire: incomplete questionnaires will be regarded as invalid; the Software EpiData version 3.1 was used for double input.

### 2.5 Statistical analysis

Excel was used to integrate the questionnaire data, SPSS version 23.0 was used for statistical description, chi-square (*X*^2^) test was used for bidirectional disordered classification data, and rank sum test was used for rank data. Statistical significance level was set at *α* = 0.05.

## 3 Results

### 3.1 Constitute of oral medical and health institutions

The number of non-public oral health institutions was 2.37 times that of public oral health institutions. Among all types of oral health institutions, the number of dental clinics was the highest, accounting for 80.3% of non-public oral health institutions and 70.4% of primary oral health institutions (Table 1).

**Table 1:** Proportion of different types of oral health institutions.

Project	Number of organizations (754)	Percentage (%)
Nature of oral organization		
Public	224	29.7
Non-public	530	70.3
Type of oral institution		
Dental specialty hospital	4	0.5
Oral outpatient department	90	11.9
Dental clinic	426	56.5
Department of Stomatology of General Hospital	113	15.0
Other hospitals’ stomatology department	32	4.3
Community health service center or township Health center Department of Stomatology department	89	11.8

### 3.2 Participation of older adults in oral medical and health institutions

Among the main payment methods for older adult patients with oral diseases in oral health institutions, the proportion of public oral health institutions’ employee medical insurance (28.6%) and urban and rural residents’ basic medical insurance (42.4%) are much higher than those of non-public oral health institutions. The main payment method for oral diseases among older adults in non-public oral health institutions was their own expense (91.3%). No public/non-public oral health institutions reported that commercial insurance was the main payment method for oral diseases in older adults. The main reasons for older adult patients seeking oral treatment at public oral health institutions are dental decay and periodontal disease, whereas at non-public oral health institutions, they are dental decay and denture restoration (Table 2).

**Table 2:** Treatment status of older adults in oral health institutions.

Project	Public [*n*(%)]	Non-public [*n*(%)]	*Z/X^2^*	*P*
Proportion of older adult patients in oral outpatient department last year			−1.08	0.28^a^
<25%	114 (50.9)	255(48.1)		
25–50%	98(43.7)	227(42.8)		
>50%	12(5.4)	48(9.1)		
The main reasons for older adult patients seeking oral treatment at the institution last year			42.63	<0.001^b^
Dental decay	93(41.5)	217(40.9)		
Periodontal disease	85(37.9)	105(19.8)		
Oral mucosal diseases	4(1.8)	3(0.6)		
Denture restoration	42(18.8)	205(38.7)		
The most important way to pay medical expenses for oral diseases of the older adult in our institution last year			339.77	<0.001^c^
UEBMI	64(28.6)	42(7.9)		
URRMI	95(42.4)	4(0.8)		
OOP	65(29.0)	484(91.3)		
Commercial insurance*	0(0)	0(0)		
Average cost of visits for oral diseases of the older adult in the previous year			−0.46	0.64^d^
≤100 yuan	54(24.1)	139(26.2)		
101–500 yuan	143(63.8)	300(56.6)		
501–1,000 yuan	19(8.4)	70(13.2)		
>1,000 yuan	8(3.5)	21(4.0)		
Per capita cost of older adult patients with oral diseases in the previous year			−1.75	0.08^e^
≤500 yuan	120(56.6)	245(46.2)		
501–1,000 yuan	82(36.6)	226(42.7)		
1,001–5,000 yuan	20(8.9)	51(9.6)		
>5,000 yuan	2(0.9)	8(1.5)		

Note: ① *The number of commercial insurance cases in public/non-public dental health institutions is 0, and the chi square analysis is not included here.

② Rank sum test for a, d, e; Fisher exact test for b; *X*^2^ test for c.

③ UEBMI, Urban Employee Basic Medical Insurance; URRMI, the Urban and Rural Resident Health insurance; OOP, out-of-pocket.

### 3.3 CME needs of oral institution regarding oral diseases in the older adult

A total of 32.8% of the medical staff in oral health institutions participated in CME on oral health-related aspects of older adults, and 69.9% of the oral health institutions had CME needs in oral health-related aspects of older adults (Table 3).

**Table 3:** Continuing medical education (CME) needs for oral diseases in the older adult in oral health institutions.

Project	Public [*n*(%)]	Non-public [*n*(%)]	Total [*n*(%)]
Whether the institution’s medical staff engaged in CME regarding oral health for older adults during the previous year			
Yes	51(22.8)	196(37.0)	247(32.8)
No	173(77.2)	334(63.0)	507(67.2)
Does the institution have CME needs related to oral health of older adults			
Yes	173(77.2)	354(66.8)	527(69.9)
No	51(22.8)	176(33.2)	227(30.1)
Do medical institutions offer a specialized geriatric oral healthcare department			
Yes	6(2.7)	14(2.6)	20(2.7)
No	218(97.3)	516(97.4)	734(97.3)

## 4 Discussion

The survey revealed that non-public oral health institutions account for a significant proportion, and the older adult primarily pay out-of-pocket for oral disease treatment at these facilities. The high cost of treatment may pose a barrier for the older adult seeking dental care. The survey also indicated that the majority of dental institutions believe there is a need to CME training for healthcare workers on the topic of geriatric oral treatment. However, the actual proportion of dental healthcare workers who have participated in CME training focused on geriatric treatment is relatively low.

China’s social basic medical insurance (SBMI) was historically split into the two components of the Urban and Rural Resident Health Insurance (URRMI) and the Urban Employee Basic Medical Insurance (UEBMI). According to the 2022 Statistical Bulletin for the Development of Medical Security, released by the National Healthcare Security Administration in 2023, SBMI coverage in China has remained above 95 percent through the end of 2022 (16). Although China has achieved universal coverage of SBMI, the designated hospitals for UEBMI and URRMI remain dominated by public medical and health institutions (16). Meng’s study (17) showed that of the 64.1% of the population covered by URBMI/NRCMS, only 5.52% used dental insurance at the time of dentist visits. The utilization rate of dental insurance for 32.8% of the population on URBMI is 13.13%. Consequently, a large number of non-public oral health institutions have not been included in the designated medical insurance units, resulting in limited coverage of SBMI for the diagnosis and treatment costs of oral diseases. This is an important reason for the high out-of-pocket payment ratio and also a significant barrier for the older adult in accessing dental services. It often necessitates self-payment when they receive services at non-public dental health institutions.

For the older adult population, higher oral healthcare expenditures impact their utilization of dental services. The prevalence rate of oral diseases in the older adults is high, but the utilization rate of oral health services is low (18). The utilization rate of oral health services for older adults aged 65–74 years is only 20.1% (7). The usage rates of oral services among the older adults are 32.6 and 39% in Brazil and North-West England, respectively. By contrast, the usage rate of oral services among the older adults in China is at a relatively low level, and needs to be improved. Previous studies have shown that older adults have high proportion of unmet oral health and that cost is still a factor in not seeking oral care, affecting the usage of oral services (19). Our survey found that the per-capita cost for older adult patients was over 500 yuan in half of oral health institutions, most of which are self funded. According to the per-capita medical and healthcare expenditures of urban and rural residents in the Guangxi Zhuang Autonomous Region in 2019, which were 1699.3 yuan and 1,088 yuan (20) respectively, the per-capita oral health expenditure of the older adults accounted for 29.4 and 46% of the per-capita medical and health care expenditures of urban and rural residents, respectively. This indicates that at least half of the older population accounted for a large proportion of the oral health expenditure. The burden of medical expenses for oral diseases in the older population is relatively heavy, which may be an important factor affecting medical treatment in the population.

Continuing medical education (CME) refers to the ongoing professional development that healthcare professionals undertake after completing their basic medical education and postgraduate training (21). It is designed to help them continuously acquire new knowledge, skills, and attitudes. Health care professionals engage in CME to ensure they remain competent to deliver high-quality, evidence-based care that supports positive patient and population health outcomes (22). Our survey indicates that 69.9% of dental institutions believe there is a need to strengthen CME training for dental healthcare workers on the topic of geriatric oral treatment. However, the actual proportion of dental healthcare workers who have participated in CME training focused on geriatric oral treatment is low, at only 32.8%. Given that the main causes of oral treatment sought by the older adult population are dental caries, periodontal diseases, and denture repair, it is recommended to establish more CME programs targeting oral diseases in the older adult, especially these common issues. This would enhance the treatment capabilities of dental healthcare workers for oral diseases in the older adult, thereby promoting positive improvements in the oral health of the older adult population.

This study has some limitations. First, this survey only investigated the data from dental medical institutions in Guangxi Zhuang, and there were no data from other provinces. Second, this was a cross-sectional survey, that may have been biased in terms of recall and reporting. Therefore, these findings should be interpreted with caution. Third, based on the perspective of oral health institutions, this study mainly conducted a descriptive analysis of the older adults’ oral disease visits and did not analyze the influencing factors. In future works, a multi-stage stratified cluster sampling method will be used to investigate the current situation, needs, and willingness to seek medical treatment for oral health care among the older adults. This will hopefully help us to better analyze the factors affecting oral health in the older population of Guangxi, and explore how to better serve the growing oral health needs of this population with dedicated oral health medical resources.

## 5 Conclusion

Our research can provide some references for policymakers in improving the oral health of the older adult population. Based on the analysis of survey results, we suggest that incorporating non-public oral health institutions that meet certain standards into the implementation scope of the Guangxi medical insurance policy, and increasing medical insurance support to expand the coverage of dental treatment insurance, can reduce the out-of-pocket rate for the older adult seeking oral health services. This would decrease the expenditure on dental treatment, enhance the utilization of dental services, and promote oral health among the older adult population. Furthermore, increasing CME programs targeted at geriatric oral diseases can significantly improve the treatment capabilities of dental healthcare workers. This would encourage them to apply more dental medical evidence in the treatment of older adult patients, effectively improving the oral health outcomes of the older adult population.

## Data Availability

The raw data supporting the conclusions of this article will be made available by the authors, without undue reservation.
